# ADH7 and plasma proteins mediate smoking-related esophageal cancer: genetic causality and therapeutic insight

**DOI:** 10.3389/fmed.2026.1745748

**Published:** 2026-10-05

**Authors:** Liangbin Pan, Xiangzhe Yang, Qingxu Hao, Kai Xie, Haitao Ma

**Affiliations:** 1 Department of Thoracic Surgery, The First Affiliated Hospital of Soochow University, Suzhou, China; 2 Department of Thoracic and Cardiovascular Surgery, Suzhou Dushu Lake Hospital, Suzhou Key Laboratory of Chest Disease Diagnosis and Treatment, The Fourth Affiliated Hospital of Soochow University, Medical Center of Soochow University, Suzhou, China

**Keywords:** biomarkers, causal relationship, esophageal cancer, plasma proteins, smoking

## Abstract

**Background:**

Smoking has been linked to alterations in plasma protein profiles and increased risk of esophageal cancer (EC). However, the precise mediating roles of plasma proteins in this relationship remain unclear. This study explores the mediation effects of plasma proteins in smoking-associated EC, utilizing genetic proxies to uncover potential biological mechanisms underpinning this association.

**Methods:**

Genetic correlation and Mendelian randomization (MR) analyses were applied to examine the association between smoking traits and EC. Pathway enrichment analysis explored circulatory protein changes in smokers. A two-step mediation MR analysis identified key plasma proteins involved in smoking-related EC pathogenesis. Survival analysis assessed the impact of transcriptional levels of mediators on EC prognosis, while phenome-wide association studies (PheWAS) evaluated the safety of targeted molecules and investigated potential pleiotropy. The expression and function of ADH7 were evaluated by qRT-PCR and *in vitro* experiments.

**Results:**

Genetic correlation analysis showed significant links between smoking and EC, supported by MR findings that confirmed a causal relationship. Smoking was found to influence pathways related to cytokine signaling, immune activation, and the STAT pathway. Five plasma proteins (ADH7, CPXM1, FUT3, GZMM, LYPD3) were identified as mediators in smoking-driven EC risk. Among these, ADH7 emerged as a key protein and was consistently associated with smoking traits; high ADH7 mRNA expression in EC tumor tissue was associated with reduced progression-free survival. PheWAS inferred that ADH7-targeted interventions might pose risks of neurological or psychiatric side effects, though these were not statistically significant. qRT-PCR and immunohistochemistry showed that ADH7 was downregulated in EC tissues relative to adjacent normal mucosa, and functional assays demonstrated that overexpression of ADH7 suppressed the proliferative and migratory capacity of EC cells. Serum ELISA further revealed that circulating ADH7 concentrations were elevated in smokers and in EC patients, with the highest levels observed in smoking EC patients, supporting a role for smoking-driven elevation of circulating ADH7 in EC risk.

**Conclusions:**

This study reveals a compartment-specific, dual role for ADH7 in smoking-related EC: elevated circulating ADH7, driven in part by smoking, is associated with increased EC risk, whereas ADH7 acts as a tumor suppressor within esophageal tumor tissue. These findings provide novel genetic and mechanistic insights into smoking-related EC pathogenesis and highlight the importance of distinguishing circulating from tissue-level protein function when interpreting biomarker-disease associations.

## 1 Introduction

Esophageal cancer (EC) represents a prevalent and highly lethal malignancy globally. As per global cancer statistics reported in 2022, there were 510,716 new cases of esophageal cancer worldwide, positioning it as the 11th most common cancer. It resulted in 445,129 deaths, making it the 7th leading cause of cancer-related mortality (1). By 2040, the global incidence of esophagus cancer is projected to increase by 63.5%, with associated deaths expected to rise by 68% (2). Despite improvements in survival rates following surgery and other treatments, the 5-year survival rate for EC remains below 25% (3), posing a significant public health challenge. Therefore, identifying, investigating, and addressing potential risk factors is crucial for improving the prognosis of esophagus cancer.

Smoking is a well-established major risk factor for EC. Tobacco contains various carcinogens, including aromatic amines, aldehydes, phenols, and nitrosamines, which can damage the esophagus mucosa and promote the initiation and progression of EC (4). Studies have demonstrated that smoking significantly alters plasma protein composition, particularly affecting fibrinogen. There is a positive correlation between the number of cigarettes smoked daily and plasma fibrinogen levels, with smoking cessation quickly reducing these levels (5–7). Fibrinogen, a key blood coagulation factor, is linked to cancer development and progression. Plasma fibrinogen levels are significantly higher in patients with malignant tumors than those with benign tumors, and this trend is also seen in patients with metastatic or advanced-stage cancer (8). This suggests that smoking-induced changes in plasma proteins may play a role in tumorigenesis and cancer progression.

Mendelian randomization (MR) is an epidemiological method that employs genetic variants from genome-wide association studies (GWAS) as instrumental variables (IVs) to explore causal relationships between exposures and diseases (9). MR can help determine whether associations observed in observational studies are causal. Unlike observational studies, MR analyses reduce confounding biases since genetic variants are randomly assigned at conception and are less susceptible to confounding factors. With the increased availability of large-scale protein quantitative trait loci (pQTL) and GWAS data, exploring causal relationships between complex traits is well-supported by robust data resources.

In this study, we employed MR analysis to explore the potential causal relationship between smoking and EC, aiming to identify plasma protein biomarkers that might play pivotal roles in EC among high-risk populations. Our findings provide new biological insights into the pathogenic mechanisms of EC in smokers and offer valuable information for developing targeted therapeutic strategies for high-risk individuals.

## 2 Methods

### 2.1 Study design and data source

We implemented a comprehensive series of analytical strategies to uncover the genetic associations between smoking and EC, as well as the pathogenic pathways mediated by plasma proteins. The study design flowchart is presented in Figure 1. The smoking-related GWAS data included Lifetime Smoking and Smoking Initiation. The Lifetime Smoking Index was derived from a GWAS study involving 462,690 individuals in the UK Biobank, while the Smoking Initiation data were obtained from a large meta-analyzed GWAS study. EC GWAS data were sourced from FinnGen R11, and plasma protein quantitative trait loci (pQTL) data were obtained from the deCODE study. All data are from European populations and are publicly available; therefore, no ethical approval or patient consent was required. Detailed data information can be found in Supplementary Table 1.

**Figure 1:**
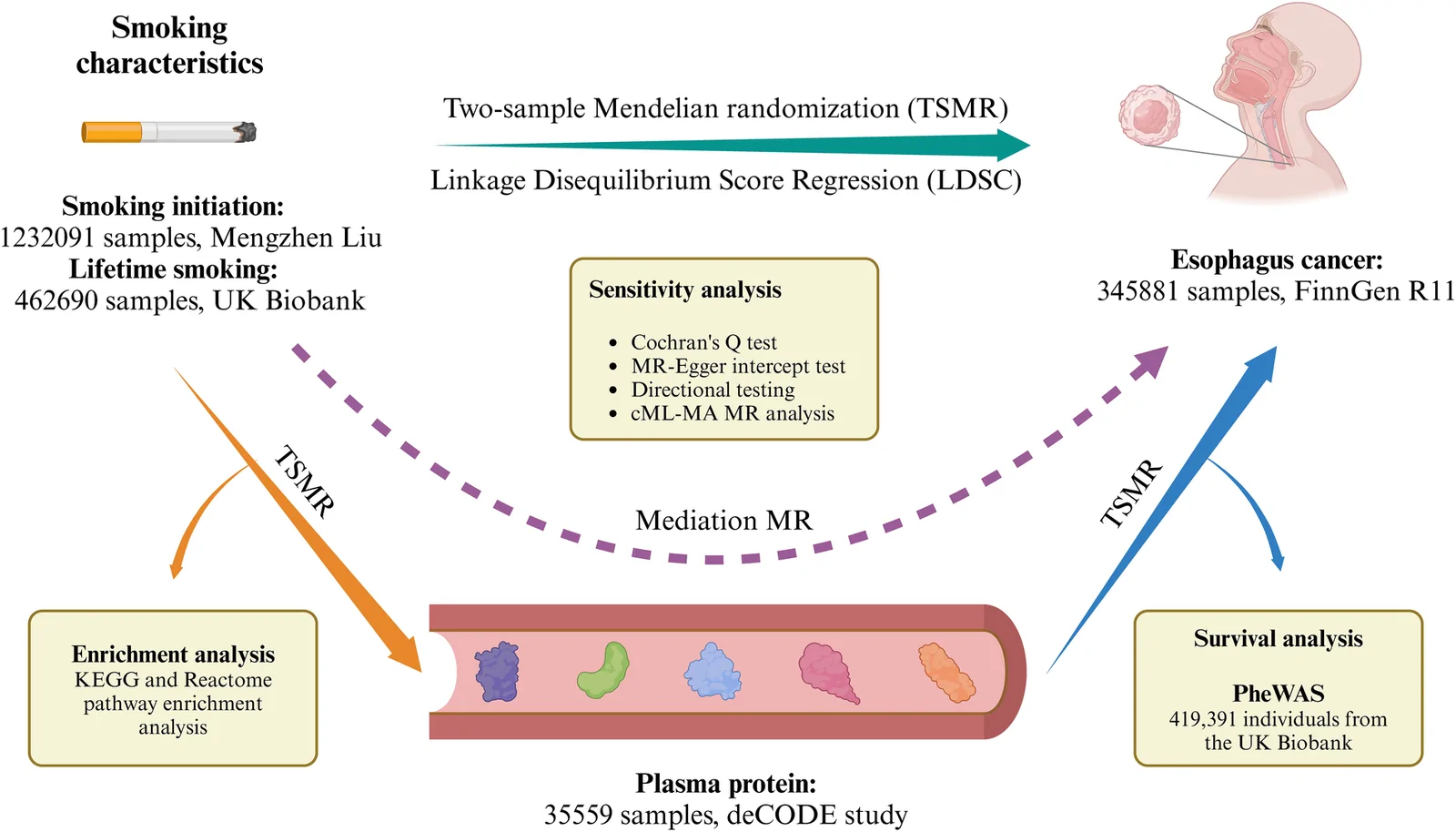
Flow chart of this study. TSMR, two-sample Mendelian randomization; LDSC, linkage disequilibrium score regression; cML-MA, constrained maximum likelihood and model averaging; KEGG, Kyoto Encyclopedia of Genes and Genomes; PheWAS, phenome-wide association study.

### 2.2 Linkage disequilibrium score regression (LDSC)

The LDSC is a widely used statistical method for evaluating genetic correlations between different phenotypes (10). By analyzing linkage disequilibrium patterns in GWAS data, it calculates the genetic correlation coefficients between traits. In this study, we employed the integrated algorithm in the R package GenomicSEM to estimate the genetic correlations between smoking traits and EC (11). This step provides essential preliminary information for subsequent causal analyses, helping to reveal potential genetic links between smoking and EC.

### 2.3 Mendelian randomization

A two-sample Mendelian randomization (TSMR) analysis was performed using the TwoSampleMR R package with default parameters, utilizing data from separate study populations to minimize potential bias due to sample overlap. The selection criteria for instrumental variables (IVs) prior to MR analysis were as follows: 1) minor allele frequency greater than 0.01; 2) single nucleotide polymorphisms (SNPs) strongly associated with the traits (*P* < 5e-8); 3) independent loci within a 1MB window with a linkage disequilibrium threshold of r^2^ < 0.001; 4) F-statistic greater than 10 (12). To reduce potential pleiotropy bias at the gene level, protein quantitative trait loci (pQTL) IVs for plasma proteins were limited to loci within 1MB upstream and downstream of the gene, excluding SNPs in the major histocompatibility complex region (Chr6, 26-34MB) (13).

Cochran's *Q* test and the MR-Egger intercept test were employed to detect potential heterogeneity and pleiotropy, using *P* < 0.05 as the threshold for statistical significance to indicate heterogeneity or horizontal pleiotropy. The inverse variance weighted (IVW) method with a random-effects model was used to mitigate bias due to SNP heterogeneity. Directional testing was also applied to rule out potential reverse causality, with “TRUE” indicating the correct direction of the MR analysis. After harmonization, the Wald ratio method (for a single IV) and the IVW method (for multiple IVs) were used as the primary analytical methods.

To evaluate the MR analysis for smoking and EC, we also employed the MR Egger, Maximum Likelihood, and Weighted Median methods as complementary approaches to assess the robustness of the genetically predicted causal associations. Additionally, the combining constrained maximum likelihood and model averaging (cML-MA) method was used to control for potential horizontal pleiotropy (14). We applied two goodness-of-fit (GOF) tests to select the optimal computation approach. If either test showed *P* < 0.05, the Bayesian Information Criterion-data perturbation (BIC-DP) method was used; otherwise, the BIC method was applied. Results were considered robust when other MR analyses yielded findings consistent with the directionality indicated by the IVW/Wald ratio method.

### 2.4 Enrichment analysis

The OmicShare website (https://www.omicshare.com/tools/) was used for Kyoto Encyclopedia of Genes and Genomes (KEGG) and Reactome pathway enrichment analyses and to visualize the results for plasma protein biomarkers related to smoking. The aim was to thoroughly explore the effect of smoking on the potential biological functions of these plasma protein biomarkers.

### 2.5 Phenome-wide association study (PheWAS)

We conducted a PheWAS using data from 419,391 individuals of European ancestry from the UK Biobank (15). This study aimed to explore the relationships between protein-coding genetic variants and various binary and continuous phenotypes. To reduce the likelihood of false positives, we adhered to the guidelines of Wang et al. (16), applying a suggestive significance threshold of 1e-6 to assess the associations between protein expression levels and phenotypes.

### 2.6 Patient tissue samples

From 2021 to 2024, twenty paired samples of EC tissues and matched adjacent non-tumor tissues were obtained from patients diagnosed with EC in the Department of Thoracic Surgery at the First Affiliated Hospital of Soochow University. Clinical information related to these patients was retrieved from their medical records. The study was approved by the Ethics Committee of the First Affiliated Hospital of Soochow University, and written informed consent was obtained from all participants. This study was conducted in accordance with the ethical principles of the World Medical Association's Declaration of Helsinki, as published in the British Medical Journal on July 18, 1964, and its subsequent amendments.

### 2.7 Cell lines

The cell lines used in this study were obtained from the Shanghai Institute of Biological Sciences (China). EC cell lines (KYSE140, KYSE150, TE-1, and TE-13) were maintained in RPMI-1640 medium (KeyGene, Nanjing, China), and human esophageal epithelial cells (HET-1A) were cultured in DMEM supplemented with 10% fetal bovine serum. All cells were incubated at 37 °C in a humidified atmosphere containing 5% CO₂.

### 2.8 RNA extraction and qRT-PCR

Total RNA was extracted from tissue samples or cells using TRIzol reagent (Invitrogen, Carlsbad, CA, USA) according to the manufacturer's instructions. Reverse transcription was performed using the PrimeScript RT kit (Takara, China), and quantitative real-time PCR (qRT-PCR) was conducted with the SYBR Select Master Mix (Thermo Fisher, China). Each qRT-PCR reaction was run in triplicate under the following cycling conditions: initial denaturation at 95 °C for 10 min, followed by 40 cycles of 95 °C for 15 s and 60 °C for 1 min. The primer sequences utilized for qRT-PCR analysis were as follows: β-actin forward, 5′-*GTCATTCCAAATATGAGATGCGT*-3′; β-actin reverse, 5′-*GCATTACATAATTTACACGAAAGCA*-3′; ADH7 forward, 5′-*ACATGAGGCAACTGGGATTGT*-3′; ADH7 reverse, 5′-*CATTGCATTCTCTACATTGTGGC*-3′.

### 2.9 Colony formation assay

Cells were trypsinized, counted, and seeded into 6-well plates at 500–1,000 cells per well in complete medium. After even distribution, cultures were maintained for 10–14 days at 37 °C in 5% CO₂, refreshing medium every 2–3 days. Colonies were washed with PBS, fixed with 4% paraformaldehyde for 15 min, and stained with 0.1% crystal violet for 20 min. Plates were rinsed, air-dried, and colonies (≥50 cells) were counted.

### 2.10 Wound healing assay

Cells were grown to a confluent monolayer in 6-well plates and serum-starved (0%–1% FBS) for 6–12 h. A straight scratch was made with a sterile 200-µL tip, and debris was removed with PBS washes. Cells were incubated in serum-reduced medium at 37 °C in 5% CO₂. Images were captured at 0 h and indicated time points (24 h, 48 h), and wound closure was quantified using ImageJ.

### 2.11 CCK-8 assay

TE-13 cells transfected with OE-ADH7 or NC were plated in 96-well plates at a density of 2 × 10^3^ cells/well and allowed to adhere for 6–8 h to establish baseline readings. At the same time point on each of four consecutive days, 10 μL of CCK-8 reagent was added to each well, followed by an additional 2-h incubation at 37 °C. Cell viability at each time point was determined by measuring absorbance at 450 nm using a microplate reader, and proliferation curves were subsequently generated based on the daily absorbance values.

### 2.12 Cell transfection

The ADH7 overexpression plasmid (OE-ADH7) and its corresponding empty vector control (NC) were transfected into TE-13 cells using Lipofectamine 3000 (Invitrogen, USA) according to the manufacturer's instructions. Briefly, cells were seeded in 6-well plates and allowed to reach approximately 70%–80% confluence prior to transfection. Plasmid DNA and Lipofectamine 3000 were separately diluted in Opti-MEM medium, combined, and incubated at room temperature for 15 min to allow complex formation. The mixture was then added dropwise to the cells, and after 6 h, the medium was replaced with complete growth medium. Cells were harvested 48 h post-transfection for subsequent validation of ADH7 expression by qRT-PCR, and successfully transfected cells were used for downstream functional assays.

### 2.13 Statistical analysis

To account for multiple testing, we used the False Discovery Rate (FDR) method, considering a corrected *P*-value of less than 0.05 (two-tailed) as statistically significant. We employed a two-step mediation approach alongside the Bootstrap method with 1,000 resampling iterations to calculate mediation (indirect) effects and assess their significance. Given the exploratory nature of high-dimensional proteomic mediation analysis, FDR correction was applied to the upstream association analyses between smoking traits and plasma proteins to control for multiple testing across the proteome. However, for the subsequent two-step mediation analysis, a nominal *P*-value threshold of <0.05 was used to identify candidate mediators, consistent with common practice in exploratory mediation screening where overly stringent correction may obscure biologically plausible signals warranting downstream functional validation. A total of 801 mediation tests were performed (53 proteins associated with Lifetime Smoking and 748 proteins associated with Smoking Initiation), and none of the identified mediators survived FDR correction at this stage. The proportion of the mediation effect was determined by dividing the mediation effect value by the total effect value. Survival curves were produced using the Kaplan–Meier Curve, gene expression was grouped based on the optimal cutoff value, and Hazard Ratio values were calculated using the Cox proportional hazards model. All statistical analyses were performed using R software (version 4.3.2).

## 3 Results

### 3.1 Relationship between smoking traits and EC risk

The information for the IVs associated with the two smoking traits is presented in Supplementary Table 2, with F-statistics ranging from 29.800 to 172.802, indicating a low likelihood of weak instrument bias in the TSMR analysis. LDSC results revealed significant genetic correlations between the smoking traits and EC (Lifetime smoking: r_g = 0.224, SE = 0.090, *P* = 0.012; Smoking initiation: r_g = 0.259, SE = 0.099, *P* = 0.009) (Table 1). Furthermore, the IVW analysis showed that higher genetically predicted Lifetime Smoking Index and Smoking Initiation were associated with an increased risk of EC (Lifetime smoking: OR = 6.570, 95% CI = 2.375–18.177, *P* = 2.883e-4; Smoking initiation: OR = 1.706, 95% CI = 1.043–2.791, *P* = 3.348e-2). The MR Egger, Maximum Likelihood, and Weighted Median methods yielded results consistent with the direction observed in the IVW analysis (Figure 2A). Cochran's Q statistics indicated a low possibility of heterogeneity across different instrumental variables (all *P* > 0.05). However, the MR-Egger intercept test suggested potential horizontal pleiotropy in the TSMR analysis of Smoking Initiation and EC (*P* = 0.008) (Supplementary Table 3). After controlling for potential pleiotropy using the cML-MA method, both Lifetime Smoking and Smoking Initiation remained significantly and causally associated with EC. Funnel plots and leave-one-out analyses for the association between smoking traits and EC risk are shown in Figures 2B,C and Supplementary Figures 1, 2. Tests for reverse causality did not provide statistically significant evidence for reverse causality between smoking and EC traits.

**Table 1:** Linkage disequilibrium score regression analysis results of smoking characteristics and esophagus cancer.

Exposure	Outcome	Correlation Coefficient (rg)	Se	Pval
Lifetime smoking	EC	0.224	0.090	0.012
Smoking initiation	EC	0.259	0.099	0.009

EC, esophagus cancer; rg, genetic correlation coefficient.

**Figure 2:**
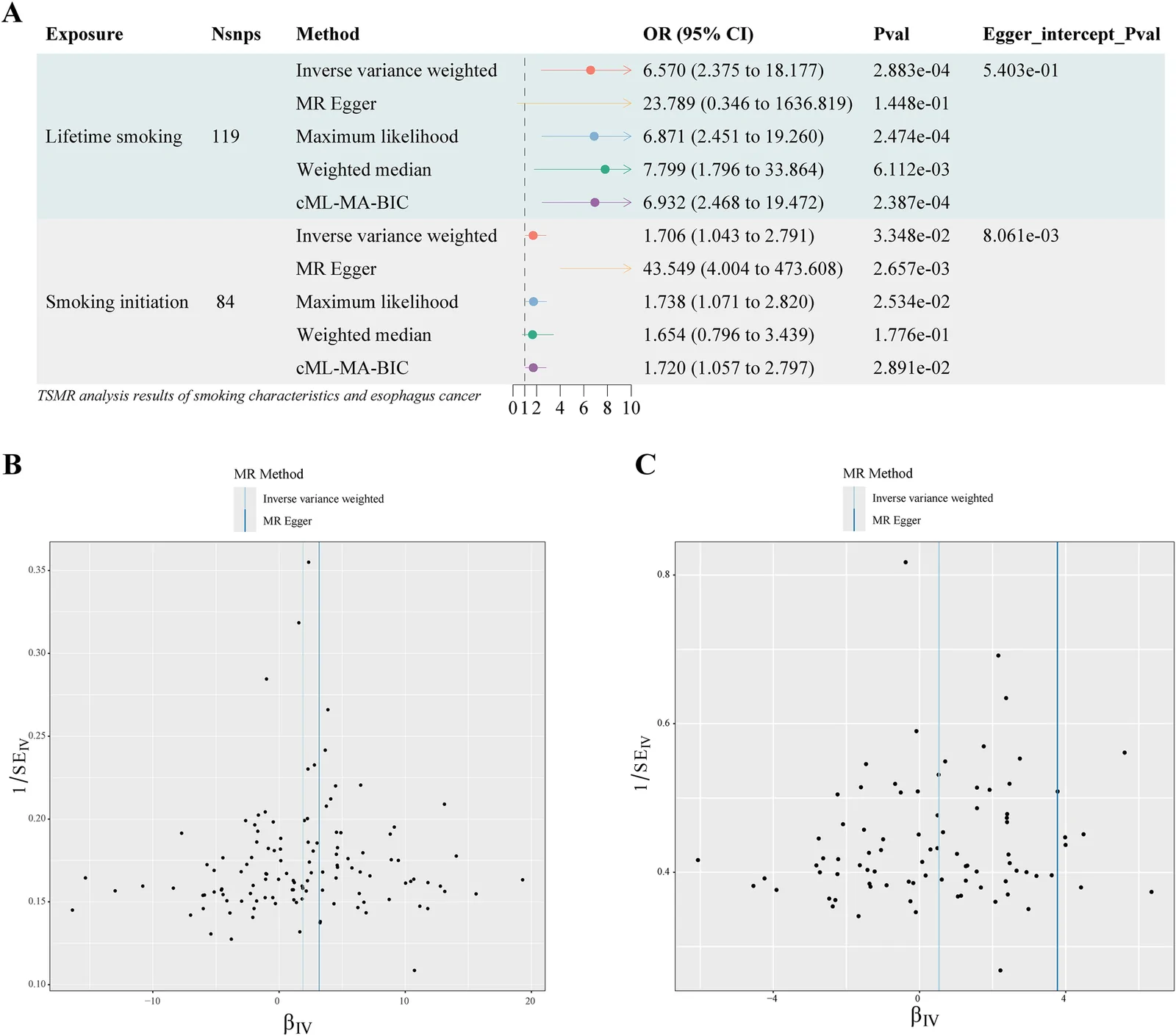
TSMR analysis results of smoking characteristics and esophagus cancer. **(A)** Forest plots of causal association between smoking and esophagus cancer. **(B)** Funnel plots of TSMR analysis between smoking initiation and esophagus cancer. **(C)** Funnel plots of TSMR analysis between lifetime smoking and esophagus cancer. TSMR, Two-sample Mendelian randomization; cML-MA, constrained maximum likelihood and model averaging; BIC, Bayesian Information Criterion.

### 3.2 Impact of smoking traits on plasma proteins

We conducted a comprehensive statistical analysis to investigate the potential impact of smoking on plasma proteins. Under the criteria of *P* < 0.05 (FDR correction), excluding reverse causality, filtering out effects with Beta < 0, and removing positive pleiotropy, we identified 53 downstream plasma protein markers associated with Lifetime Smoking and 748 markers associated with Smoking Initiation (Supplementary Tables 4, 5). To further explore the biological significance of these smoking-affected circulating plasma proteins, we performed pathway enrichment analyses using the KEGG and Reactome databases. The results indicated that these plasma proteins were significantly enriched in cytokine signaling, STAT pathways, and immune-related signaling pathways (Figures 3A,B).

**Figure 3:**
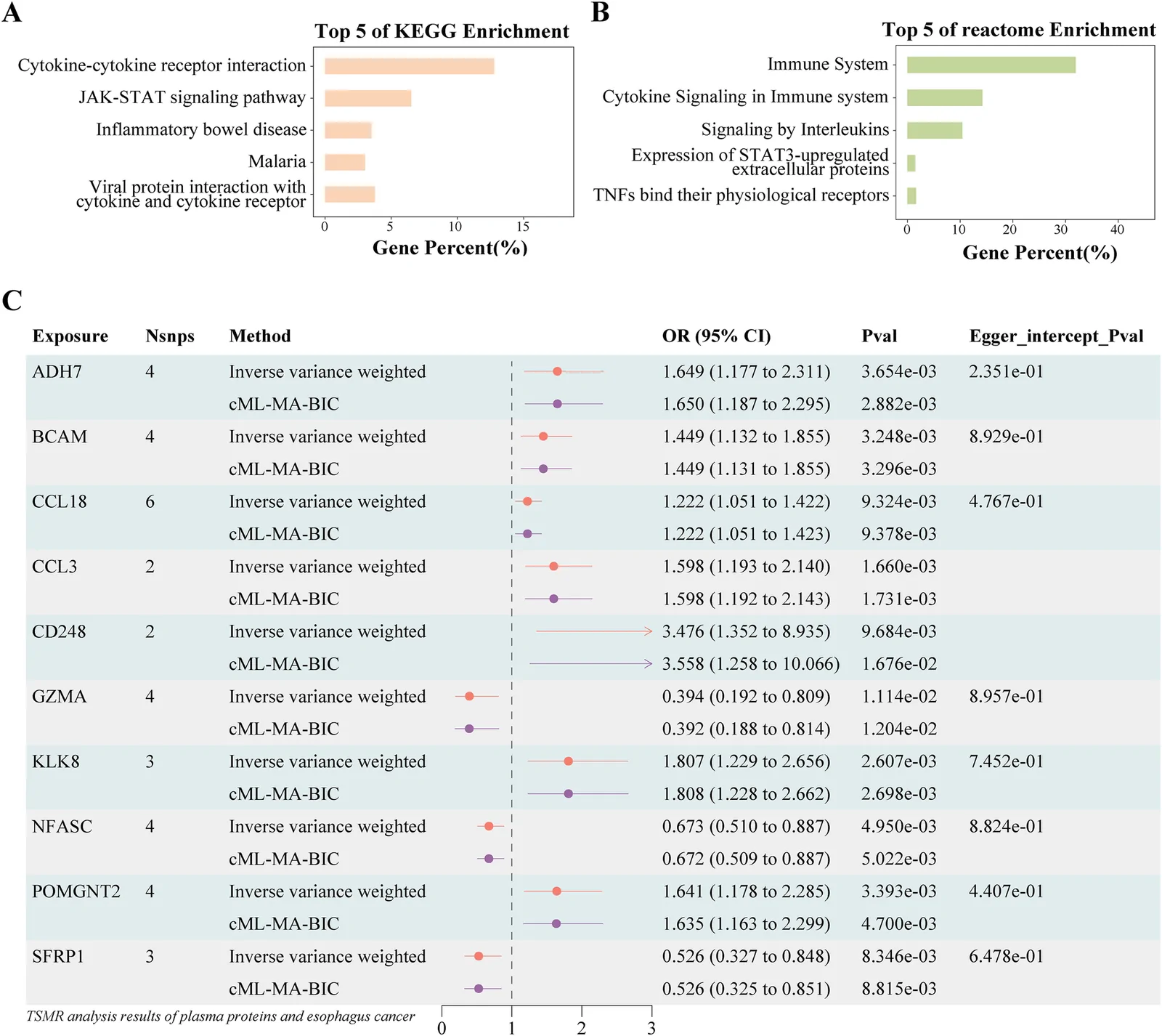
Causal pathogenic proteins in esophagus cancer and enrichment analysis of smoking altered plasma proteins. **(A)** KEGG enrichment analysis of smoking altered plasma proteins. **(B)** Reactome enrichment analysis of smoking altered plasma proteins; **(C)** Forest plot of the ten plasma proteins most strongly associated with esophagus cancer by TSMR analysis. TSMR, Two-sample Mendelian randomization; KEGG, Kyoto Encyclopedia of Genes and Genomes; cML-MA, constrained maximum likelihood and model averaging; BIC, Bayesian Information Criterion.

### 3.3 Impact of plasma proteins on EC

To identify plasma proteins with potential causal associations with EC, we conducted a TSMR analysis using pQTL data from deCODE and EC GWAS data from the FinnGen study prior to mediation analysis. The IVW and Wald ratio methods identified 67 plasma proteins associated with EC risk, while the cML-MA method identified 68 potential pathogenic plasma proteins for EC. However, none of these proteins met the significance threshold after FDR correction. We present the 10 proteins with the lowest *P*-values from the IVW and Wald ratio analyses, among which higher expression levels of seven targets (ADH7, BCAM, CCL18, CCL3, CD248, KLK8, POMGNT2) were associated with an increased risk of EC, while higher expression levels of the remaining three targets (GZMA, NFASC, SFRP1) were protective against EC. After controlling for horizontal pleiotropy, the cML-MA method produced consistent positive results with the primary findings (Supplementary Table 6 and Figure 3C).

### 3.4 Mediation MR analysis

Mediation analysis was employed to identify key plasma proteins that may mediate the effects of smoking on EC development. We included all TSMR pathways in the mediation analysis to explore more potential mediators. A total of 801 mediation pathways were tested, comprising 53 plasma proteins significantly associated with Lifetime Smoking and 748 proteins associated with Smoking Initiation in our upstream analyses. After screening, five plasma proteins (ADH7, CPXM1, FUT3, GZMM, LYPD3) were found to be involved in the smoking-related formation of EC at a nominal significance threshold of *P* < 0.05, with mediation effects ranging from 1.711%–21.819% (Supplementary Table 7 and Figure 4A). The 95% confidence intervals for the indirect effects were as follows: ADH7 via Lifetime Smoking (indirect effect 0.047, 95% CI = 0.010–0.203, mediation proportion 2.480%), ADH7 via Smoking Initiation (indirect effect 0.025, 95% CI = 0.007–0.104, mediation proportion 4.648%), CPXM1 (indirect effect 0.020, 95% CI = 0.001–0.098, mediation proportion 3.830%), FUT3 (indirect effect 0.009, 95% CI = 0.000–0.052, mediation proportion 1.711%), GZMM (indirect effect 0.043, 95% CI = 0.007–0.650, mediation proportion 21.819%), and LYPD3 (indirect effect 0.043, 95% CI = 0.002–0.228, mediation proportion 7.989%). All confidence intervals excluded zero and demonstrated positive directional effects. Notably, ADH7 was repeatedly identified in the pathways linking smoking to EC. Individuals with higher levels of Lifetime Smoking and Smoking Initiation exhibited elevated plasma ADH7, which in turn increased EC risk, accounting for 2.480% to 4.648% of the total effect. The principles of mediation analysis and the mediator pathway diagram for ADH7 involvement are shown in Figure 4B.

**Figure 4:**
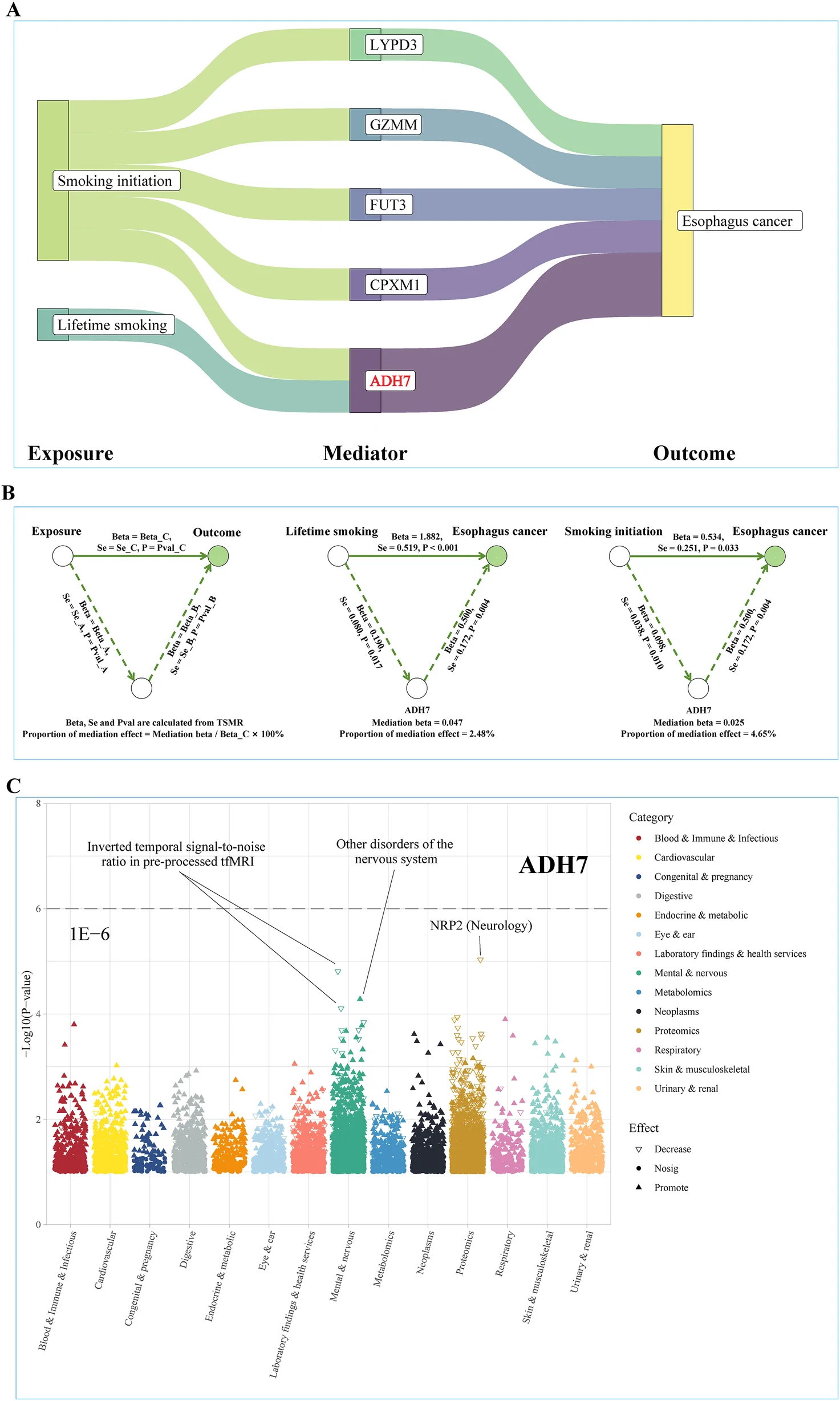
Analysis of plasma protein mediation and safety evaluation of key proteins in smoking-induced esophagus cancer. **(A)** Sankey diagram of plasma protein mediation analysis of smoking-induced esophagus cancer. **(B)** Mediation schematic diagram and mediating effects of the plasma protein ADH7 mediated by smoking characteristics. **(C)** Manhattan plot of phenome-wide association study for ADH7. TSMR, Two-sample Mendelian randomization; cML-MA, constrained maximum likelihood and model averaging; BIC, Bayesian Information Criterion.

### 3.5 Survival analysis

To further evaluate the impact of the mediating plasma proteins on the prognosis of EC, we analyzed the relationship between the transcriptional expression levels of the aforementioned five protein mediators (ADH7, CPXM1, FUT3, GZMM, and LYPD3) and the overall survival (OS) and progression-free survival (PFS) of EC patients using publicly available bulk transcriptomic data from EC tumor tissues. The results showed that high transcriptional expression of ADH7 in EC tumor tissues was significantly associated with shorter PFS, while the other molecules did not demonstrate a statistically significant association with survival outcomes in EC patients (Supplementary Figure 3). It should be noted that this prognostic analysis was based on ADH7 mRNA expression levels in tumor tissues, not on circulating ADH7 protein levels.

### 3.6 Phenome-wide association study (PheWAS)

Given that most therapeutic agents necessitate sustained systemic exposure to achieve clinical efficacy, we conducted a PheWAS to systematically assess potential associations between genetically proxied ADH7 expression and a broad range of binary and continuous phenotypes. This analysis serves as a proxy for chronic systemic exposure, aiming to reveal potential drug toxicity and side effects for key therapeutic targets, as well as pleiotropy not detected by standard MR analysis. Overall, no significant associations were observed between genetically proxied ADH7 expression and any phenotype at a suggestive significance threshold (*P* < 1e-6). At a suggestive *P* threshold of 1e-4, ADH7 expression was primarily associated with neurological and psychiatric traits, including the inverted temporal signal-to-noise ratio in pre-processed task-based functional MRI (tfMRI), other disorders of the nervous system, and the plasma protein NRP2 under the neurology system category. Detailed results are provided in Supplementary Tables 8, 9 and Figure 4C.

### 3.7 Overexpression of ADH7 inhibits proliferation and migration of EC cells

Single-cell RNA-sequencing analysis of the GSE160269 dataset revealed that ADH7 expression was largely restricted to malignant epithelial cells (Figures 5A,B). Consistent with this, qRT-PCR and immunohistochemistry showed that ADH7 was markedly downregulated in EC tissues compared with paired adjacent normal mucosa at both the mRNA and protein levels (Figures 5C,D), and this reduction was also observed across EC cell lines relative to the normal esophageal epithelial cell line HET-1A (Figure 5E). To investigate the functional consequences of ADH7 downregulation, we established stable ADH7 overexpression in TE-13 cells by plasmid transfection (Figure 5F). CCK-8, colony formation, and wound-healing assays showed that restoring ADH7 expression suppressed proliferation and migration of EC cells (Figures 5G–I). We compared serum ADH7 concentrations across four groups: non-smoking healthy controls, smoking healthy controls, non-smoking EC patients, and smoking EC patients. Serum ADH7 levels differed significantly among the four groups. ADH7 concentrations were lowest in non-smoker controls, increased in smoker controls and non-smoker EC patients, and were highest in smoker EC patients (Figure 5J). Two-way ANOVA revealed significant main effects of smoking and disease status, as well as a significant smoking × disease interaction, indicating that smoking was associated with a greater increase in serum ADH7 levels in patients with EC (Figure 5J).

**Figure 5:**
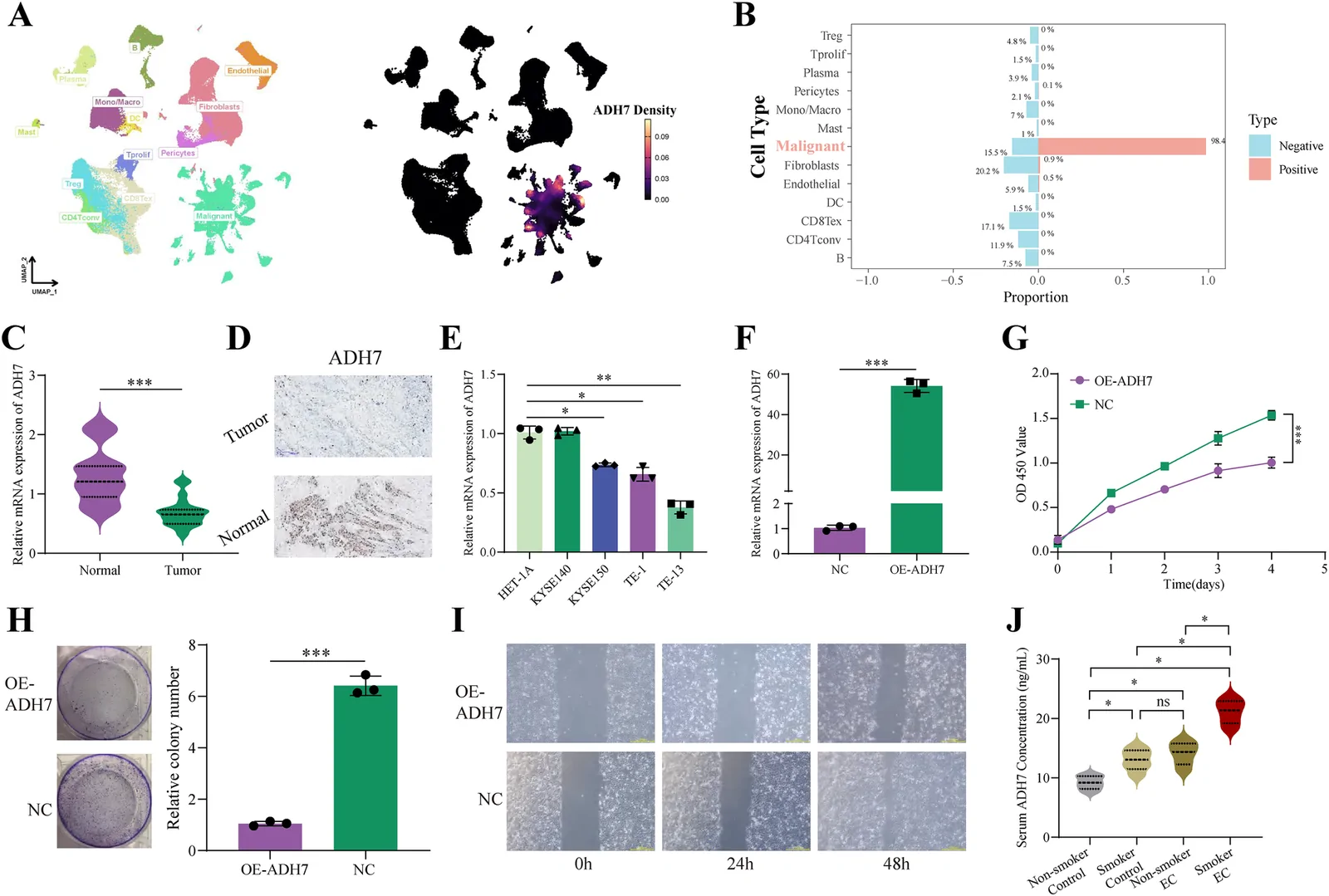
ADH7 is downregulated in EC and inhibits proliferation and migration of EC cells. **(A,B)** UMAP plots of the GSE160269 single-cell dataset showing major cell clusters **(A)** and ADH7 expression restricted mainly to malignant epithelial cells **(B)**. **(C)** qPCR showing reduced ADH7 mRNA levels in EC tissues and adjacent normal mucosa (*n* = 20). **(D)** Representative immunohistochemistry images in EC tissues compared with paired adjacent normal mucosa (scale bar, 100 μm). **(E)** qPCR showing reduced ADH7 mRNA levels in EC cell lines relative to HET-1A. **(F)** Validation of ADH7 overexpression in TE-13 cells. **(G–I)** CCK-8 **(G)**, colony formation **(H)**, and wound-healing (scale bar, 200 μm) **(I)** assays showing that ADH7 overexpression suppressed proliferation and migration of TE-13 cells. **(J)** Serum ADH7 concentrations in non-smoking controls, smoking controls, non-smoking EC patients, and smoking EC patients. Data are mean ± SD. *P < 0.05, **P < 0.01, ***P < 0.001.

## 4 Discussion

This study employed a series of advanced methodologies to elucidate the relationship between smoking characteristics and EC, as well as the potential plasma protein-mediated mechanisms in high-risk populations. The results demonstrated a strong genetic correlation between the Lifetime Smoking Index, smoking initiation, and EC. MR analysis underscored the causal role of smoking in promoting EC development. Plasma proteins in smokers exhibited alterations that predominantly enriched in inflammation-related signaling pathways. Mediation analysis identified five potential protein mediators through which smoking contributes to EC development: ADH7, CPXM1, FUT3, GZMM, and LYPD3. Notably, ADH7 was consistently involved in the development of EC across both smoking characteristics, and high ADH7 expression in EC tumor tissue was associated with shorter progression-free survival in EC patients. Subsequent analyses suggested that targeted therapy against ADH7 might be associated with neuropsychiatric adverse effects, although this association did not reach statistical significance.

The physiological environment of smokers undergoes significant alterations due to long-term exposure to toxic substances, which not only disrupt normal biological functions but also promote tumor initiation and progression (17, 18). Numerous studies have established the pivotal role of smoking in the development and progression of EC (4). Additionally, EC patients with a history of smoking often exhibit varying sensitivities to different therapeutic approaches. Understanding the distinct mechanisms of disease development, in conjunction with patients’ lifestyle factors, including smoking habits, can facilitate the selection of personalized treatment strategies. By employing a mediation MR approach, this study uncovered novel pathogenic targets associated with smoking-induced EC. These findings not only enhance our understanding of the causal pathways between smoking and EC but also lay the groundwork for developing more targeted therapeutic interventions for smokers at high risk of EC.

Some of our findings align well with previous studies. LYPD3 is known as a highly glycosylated oncogenic cell surface protein that plays a crucial role in the tumorigenesis of various solid tumors (19). It not only promotes tumor formation but also helps maintain melanoma cell stemness and immune suppression (20). Gu et al. demonstrated that alterations in the upstream LKB1-CRTC signaling pathway of LYPD3 are associated with the malignant biological behavior of EC cells (21), consistent with part with our findings. GZMM, a unique granzyme, has been implicated in both innate and tumor immunity (22, 23). Interestingly, previous MR analysis have identified GZMM as an important carcinogenic target in the context of lifestyle factors (24). Our mediation analysis further highlighted GZMM as a key biological mediator in smoking-induced EC, with the highest mediation proportion (21.819%). In our enrichment analysis, plasma protein changes in smokers were linked to immune responses, suggesting that GZMM's role in immune-related responses may be significant in the pathogenesis of smoking-related EC.

In cancer signaling pathways, FUT3, a crucial member of the FUT family, is closely associated with the TGF-*β* signaling pathway and epithelial-mesenchymal transition (25). High expression of FUT3 has been reported in lung adenocarcinoma (26), and our study suggests a potential involvement in EC. Additionally, CPXM1, another significant gene, is known to influence the tumor immune microenvironment in head and neck squamous cell carcinoma and could potentially affect the prognosis of immune-related therapies (27). Similar implications have been suggested in gastric cancer (28), and our results indicate a potential role for CPXM1 in EC. ADH7, part of the alcohol dehydrogenase gene family, is associated with various upper aerodigestive tract cancers (29). A large study in a Japanese population showed that the rs3737482 locus of *ADH7* was more strongly associated with upper aerodigestive tract cancers in heavy smokers (30). Although our research focused on a European cohort, it provides deeper insights into the potential mechanisms of ADH family involvement in smoking-related EC.

An important consideration in interpreting our findings is the apparent divergence between circulating and tissue-level ADH7 in EC. Genetically predicted circulating ADH7 (Mendelian randomization) and directly measured serum ADH7 (ELISA) were both positively associated with EC risk, particularly in smokers, whereas ADH7 was downregulated in EC tissues and its restoration suppressed the proliferation and migration of EC cells. In addition, higher tissue ADH7 mRNA expression was associated with shorter PFS, which at first appears to contradict the tumor-suppressive phenotype observed in our functional experiments. It is important to note that the prognostic analysis was based on bulk tumor mRNA rather than circulating protein, so these two sets of observations are not directly comparable. We propose three explanations for the prognostic finding. First, it was derived from heterogeneous patient cohorts in which smoking intensity, tumor stage, treatment history, and microenvironment composition are not captured by univariable survival estimates, and it therefore cannot be equated with the intrinsic, context-controlled effect of ADH7 measured *in vitro*. Second, our single-cell analysis of GSE160269 shows that ADH7 is largely restricted to and enriched in malignant epithelial cells (Figures 5A,B); bulk tissue downregulation is therefore consistent with loss of normal epithelial architecture and dilution by stromal and immune cells, whereas within the surviving malignant compartment, relatively higher ADH7 expression may reflect tumor heterogeneity or an adaptive response in progressive disease, with a biological meaning distinct from its role during early tumorigenesis. Third, our experimental systems isolate ADH7's intrinsic activity and consistently demonstrate a tumor-suppressive effect (Figures 5F–I), which we consider the most direct readout of its function in esophageal epithelial cells. We also emphasize that the PFS association was of borderline significance (HR = 1.96, 95% CI 1.02-3.78, *P* = 0.043) and was not accompanied by a significant association with overall survival (HR = 1.85, 95% CI 0.83-4.09, *P* = 0.13); this result should therefore be regarded as exploratory and does not override the experimental evidence for a tissue-level tumor-suppressive role. Collectively, these observations support a context-dependent role for ADH7 that varies by biological compartment (circulation vs. tissue), cellular context (normal epithelium vs. malignant cells within heterogeneous tumors), and disease stage, and they underscore the importance of distinguishing circulating from tissue-level protein function when interpreting biomarker-disease associations.

This study has several important limitations that warrant careful consideration. First, we were unable to directly test the mechanism underlying the divergence between circulating and tissue ADH7 within the scope of the present study. We speculate that circulating and tissue ADH7 may be governed by distinct regulatory mechanisms—for example, circulating ADH7 may originate largely from hepatic or extra-esophageal sources and act through systemic pathways independent of its local expression in esophageal tissue—but this remains an untested hypothesis rather than a demonstrated mechanism. Our tissue-level findings are consistent with prior reports of ADH7 downregulation in EC and with genetic evidence implicating alcohol-metabolizing enzymes in EC susceptibility, which supports the tissue-level interpretation but does not by itself resolve the discrepancy with the circulating-level findings. Paired plasma-tissue sampling, mechanistic studies of ADH7 secretion, and functional work testing whether circulating ADH7 acts through a paracrine or systemic route independent of local tissue expression would be needed to clarify this relationship. Until such data are available, the biomarker and therapeutic implications of ADH7 should be interpreted with caution, given the possibility that circulating and tissue ADH7 play opposing roles. Second, our discovery phase utilized European-ancestry GWAS and pQTL datasets, while experimental validation employed an Asian clinical cohort. Rather than viewing this solely as a limitation, we propose this represents a valuable opportunity to demonstrate cross-ancestry biological conservation. The fact that ADH7-mediated pathways identified through European genetic data show consistent functional effects in Asian cellular models and correlate with clinical outcomes in Asian patients suggests that the underlying biology is likely conserved rather than a population-specific artifact. The genetic instruments we employed are based on cis-acting protein quantitative trait loci, which typically exhibit more consistent effects across ancestries compared to trans-acting or non-coding regulatory variants. However, we acknowledge that population-specific allele frequencies, linkage disequilibrium patterns, and gene-environment interactions may still influence the magnitude or penetrance of ADH7 effects in different populations. Currently, large-scale plasma pQTL resources comparable to the deCODE study are not yet available for East Asian ancestries, which precludes immediate genetic replication in an independent Asian cohort. Nevertheless, our experimental validation in Chinese clinical samples and cell lines provides interim evidence supporting the biological relevance of ADH7 across populations. Third, our study integrates evidence from multiple biological levels including genetically predicted plasma proteins, tissue mRNA expression, and cellular functional assays. While this multi-level approach provides complementary evidence, we recognize that each level addresses distinct biological questions and operates on different time scales. The genetic MR analysis establishes population-level causal relationships reflecting lifelong exposure, tissue expression analysis reveals molecular states in established disease, and functional experiments demonstrate mechanistic potential under controlled conditions. These should be viewed as converging lines of evidence that together inform the compartment-specific role of ADH7 in smoking-related EC, rather than as direct replications that must show identical directional effects. The modest sample size of our clinical cohort limits statistical power for stratified analyses and precludes robust examination of potential effect modifiers such as smoking intensity or tumor stage. Future studies incorporating larger multi-center cohorts, animal models with systemic ADH7 modulation to bridge the gap between circulating and tissue effects, direct protein quantification via immunohistochemistry and enzyme-linked immunosorbent assays in both plasma and tissues, longitudinal sampling across disease progression, and spatial transcriptomics to resolve intra-tumoral ADH7 heterogeneity will provide more definitive integration across biological levels and stronger causal evidence. Despite these limitations, our study establishes a robust genetic foundation for ADH7 as a mediator of smoking-related EC risk, demonstrates its functional relevance through experimental validation, and provides a clear framework for future mechanistic investigations that can address these remaining questions.

## 5 Conclusions

This study demonstrates that smoking is causally associated with increased EC risk, mediated in part through elevated circulating levels of plasma proteins including ADH7. While Mendelian randomization and serum ELISA data indicate that higher circulating ADH7, particularly in smokers, is associated with increased EC risk, functional experiments show that ADH7 acts as a tumor suppressor within esophageal tumor tissue, where its expression is markedly reduced and its restoration inhibits proliferation and migration of EC cells. These findings suggest that circulating and tissue ADH7 may be governed by distinct regulatory mechanisms and may exert opposing roles depending on biological compartment, a distinction that should be carefully considered before ADH7 is pursued as a therapeutic or biomarker target. Further studies, including paired plasma-tissue sampling and mechanistic investigation of ADH7 secretion and compartment-specific signaling, are needed to clarify how smoking-induced systemic elevation of ADH7 relates to its local tumor-suppressive function, and to evaluate the safety and feasibility of targeting this pathway in high-risk populations.

## Data Availability

The original contributions presented in the study are included in the article/Supplementary Material, further inquiries can be directed to the corresponding authors.

## Supplementary material

Supplementary Figure 1 Leave-one-out analyses results of Lifetime smoking and esophagus cancer. EC: Esophagus cancer.

Supplementary Figure 2 Leave-one-out analyses results of Smoking initiation and EC. EC: Esophagus cancer.

Supplementary Figure 3 Transcriptional expression of mediation plasma protein in relation to prognosis in EC.
